# Light-incubation effects on lateralisation of single unit responses in the visual Wulst of domestic chicks

**DOI:** 10.1007/s00429-021-02259-y

**Published:** 2021-03-30

**Authors:** Giacomo Costalunga, Dmitry Kobylkov, Orsola Rosa-Salva, Giorgio Vallortigara, Uwe Mayer

**Affiliations:** 1 Center for Mind/Brain Science, University of Trento, Piazza Manifattura 1, 38068 Rovereto, TN Italy; 2 Max Planck Institute for Ornithology, Seewiesen, Germany

**Keywords:** Lateralisation, Visual Wulst, Domestic chicks, Development, Light exposure, Electrophysiology

## Abstract

Since the ground-breaking discovery that in-egg light exposure triggers the emergence of visual lateralisation, domestic chicks became a crucial model for research on the interaction of environmental and genetic influences for brain development. In domestic chick embryos, light exposure induces neuroanatomical asymmetries in the strength of visual projections from the thalamus to the visual Wulst. Consequently, the right visual Wulst receives more bilateral information from the two eyes than the left one. How this impacts visual Wulst’s physiology is still unknown. This paper investigates the visual response properties of neurons in the left and right Wulst of dark- and light-incubated chicks, studying the effect of light incubation on bilaterally responsive cells that integrate information from both eyes. We recorded from a large number of visually responsive units, providing the first direct evidence of lateralisation in the neural response properties of units of the visual Wulst. While we confirm that some forms of lateralisation are induced by embryonic light exposure, we found also many cases of light-independent asymmetries. Moreover, we found a strong effect of in-egg light exposure on the general development of the functional properties of units in the two hemispheres. This indicates that the effect of embryonic stimulation goes beyond its contribution to the emergence of some forms of lateralisation, with influences on the maturation of visual units in both hemispheres.

## Introduction

Contrary to what was once believed, functional and structural differences between cerebral hemispheres (i.e., brain lateralisation) are widespread in the animal kingdom (Vallortigara and Rogers 2005; Frasnelli et al. 2012; Rogers et al. 2013). Due to the almost complete decussation of the optic nerve fibers, birds with laterally placed eyes have been often used as models for the investigation of asymmetries in visual processing. As a consequence, in avian species each eye sends the majority of its visual information to the contralateral hemisphere (Cowan et al. 1961; Butler and Hodos 2005). However, even though birds lack any structure comparable to the mammalian corpus callosum (Mihrshahi 2006), the hippocampal, anterior, tectal and posterior commissures still allow exchange of information between the two hemispheres. This is an additional layer of complexity that needs to be considered in the interpretation of visual lateralization effects in avian species (e.g., Rogers and Sink 1988; Rogers and Deng 1999 showed that lateralization affects specifically structures involved in the processing of bilateral information, see below).

Within birds, domestic chicks emerged as a particularly relevant model, after the ground-breaking finding that exposure of chick eggs to light during a specific sensitive period causes the development of visual lateralisation (Rogers 1982). Following research revealed that light exposure during the development evokes several forms of behavioural and neuroanatomical lateralisation, including asymmetries of the supraoptic decussation, a recrossing tract bringing to each hemisphere projections from its ipsilateral eye (Rogers and Sink 1988; Rogers and Deng 1999). These asymmetries are present only in chicks hatched from light-incubated eggs, but not in dark-incubated chicks (e.g., Rogers and Sink 1988; Rogers 1990, 1997; Rogers and Bolden 1991; Deng and Rogers 1997; Rogers and Deng 1999; Johnston and Rogers 1999; Andrew et al. 2004; Dharmaretnam and Rogers 2005; Daisley et al. 2010).

In the last stages of incubation, avian embryos are asymmetrically oriented in the egg, with the left eye covered by the body and the right eye facing the eggshell (Kuo 1932). When eggs are exposed to light during this sensitive period, as it often occurs in the natural environment (Buschmann et al. 2006), light stimulates only the right eye, causing the asymmetrical development of the left and right sides of the visual system (Güntürkün and Ocklenburg 2017). This anatomical asymmetry only persists for the first 3 weeks after hatching (Rogers and Sink 1988). However, a wide range of behavioural asymmetries have been found to emerge as a consequence of in-egg light exposure in domestic chicks, some of which persist into adulthood (Andrew 1991; Mckenzie et al. 1998). At the same time, behavioural lateralisation has been widely documented both in light-incubated (Vallortigara and Andrew 1991, 1994; Vallortigara 1992; Andrew et al. 2004; Dharmaretnam and Rogers 2005; Rosa Salva et al. 2007, 2012; Daisley et al. 2010; Rugani et al. 2015) and dark-incubated chicks (Vallortigara et al. 2001; Mascetti and Vallortigara 2001; Deng and Rogers 2002; Chiandetti 2011; Chiandetti et al. 2013; Chiandetti and Vallortigara 2019), revealing the presence of diverse mechanisms for the development of behavioural asymmetries.

In contrast to a well-described lateralisation of behavioural traits, only few studies have been devoted to the neural correlates of these phenomena (e.g., Rogers and Sink 1988; Rogers and Bolden 1991; Deng and Rogers 1997; Rogers and Deng 1999; Lorenzi et al. 2019). Specifically, functional lateralisation in the avian visual system and the role of light-exposure in its development have never been investigated at the level of neural response properties.

**Fig. 1:**
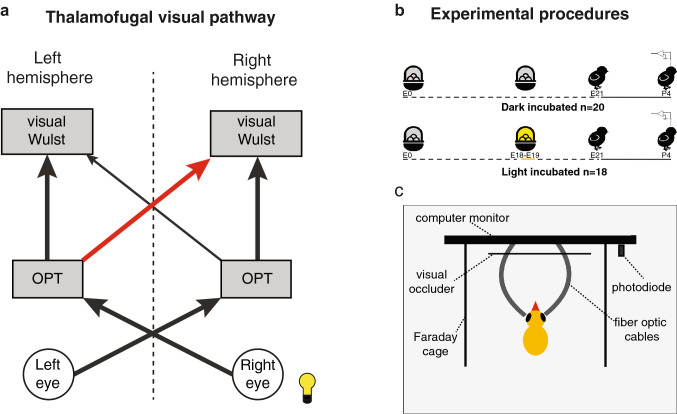
On the left (**a**), a simplified view of the thalamofugal visual pathway in domestic chicks, highlighting the light-induced asymmetry in the recrossing projections of the supraoptic decussation. The red arrow represents the stronger projections reaching the right visual Wulst from the left OPT (opticus principalis thalami), compared to those reaching the left visual Wulst from the right OPT (thinner black arrow). On the top right (**b**), a schematic representation of timeline for the light-incubation treatment of the dark and light-incubated groups. For the light-incubated group, the eggs were exposed to light during embryonic days 18 and 19 (E18–E19). Hatching took place after 21 days of incubation (E21) and recordings took place at post-hatching day 4 (P4). On the bottom right (**c**), a schema of the experimental apparatus (viewed from above). Chicks were placed on an anti-vibration table, with a computer monitor in front of them and two lateral walls acting both as a Faraday cage and as visual occluders from the external environment. An additional visual occluder was placed in front of the screen, to prevent any frontal visual stimulation. Experimental stimuli to the two eyes were provided by fibre-optic cables placed directly in front of each eye, which were conducting light from short flashing stimuli appearing on the screen. Additionally, a photodiode placed on the portion of the screen outside chicks’ view was activated by a synchronous trigger signal

The avian visual system is composed of two primary ascending visual pathways, known as the tectofugal and thalamofugal pathways (Bischof and Watanabe 1997; Clark and Colombo 2020; Knudsen 2020). In both pathways, most visual projections from each eye reach the contralateral hemisphere, due to the almost complete crossing of the optic nerve. However, both pathways send at least some visual information also to the hemisphere ipsilateral to the seeing eye, through recrossing projections. While in chicks both visual pathways present some level of asymmetries (e.g., Morandi-Raikova et al. 2021), the thalamofugal pathway, which is the focus of the present study, presents a higher degree of lateralisation (Rogers and Sink 1988; Rogers and Deng 1999; Deng and Rogers 2002). Through this visual pathway, retinal projections reach the nucleus opticus principalis thalami of the contralateral hemisphere (OPT, considered homolog of the mammalian lateral geniculate nucleus). Each OPT projects then to a structure called the visual Wulst (equivalent to the mammalian primary visual cortex) on the dorsal part of the frontal telencephalon. Even though most projections from each OPT reach its ipsilateral Wulst, a minority of recrossing projections, forming the supraoptic decussation, reach the contralateral hemisphere, providing this structure with information from both eyes (Fig. 1a).

The visual Wulst is thus the main telencephalic recipient of the thalamofugal pathway (Karten et al. 1973). The visual Wulst is composed of four main layers. The middle layer (IHA—nucleus interstitialis hyperpallii apicale) receives most of the visual projections originating from the OPT (but see Karten et al. 1973) and projects to the upper layer (HA—hyperpallium apicale). From there, projections reach back to the thalamus and to the lowest layers of the visual Wulst (HI—hyperpallium intercalatum and HD—hyperpallium densocellulare). Visual Wulst sends projections to various structures including the hippocampal formation and the nido- and mesopallium (Shanahan et al. 2013; Atoji et al. 2018). The visual Wulst contains many visually responsive neurons (Revzin 1969; Gusel’nikov et al. 1977; Ng et al. 2010), which are organised according to multiple retinotopic maps (Michael et al. 2015; Bischof et al. 2016) and are sensitive to motion and orientation akin to those found in the mammalian visual cortex. Visual Wulst also shows column-like organisation (Stacho et al. 2020). However, due to the different organisation of birds pallium, the layers of the visual Wulst do not correspond to the layers of mammalian cortex (Medina and Reiner 2000).

Even though the visual Wulst has been found to be involved in processing of visuospatial information (Watanabe et al. 2011), sun compass-based orientation (Budzynski et al. 2002) and vision-mediated earth magnetic field orientation (Mouritsen et al. 2005; Zapka et al. 2009), the functions of this structure are still only partially understood. At the functional level, the visual Wulst had been particularly well-investigated in birds with frontally placed eyes and a good degree of binocular overlap. In owls, for instance, visual Wulst neurons present precise retinotopic organisation, direction and orientation selectivity and binocular disparity (Pettigrew and Konishi 1976; Nieder and Wagner 2000). Indeed, in birds with frontally placed eyes, processing of binocular information for depth estimation seems to be one of the main functions of the visual Wulst (Nieder and Wagner 2000; Blake and Wilson 2011), whose size seems to correlate with the amount of binocular overlap of each species (Iwaniuk et al. 2008).

On the contrary, for birds with laterally placed eyes and a lower degree of binocular overlap (such as domestic chicks, but also pigeons and zebra finches), the amount of binocular information processing carried out by the visual Wulst is still unclear. Until now, in chicks, zebra finches and pigeons only a marginal binocular interaction has been demonstrated (Parker and Delius 1972; Wilson 1980; Denton 1981; Bredenkötter and Bischof 1990a, b). In these birds, bilateral integration of information may even serve different functions than it does in owls. For instance, in zebra finches, stimulation of the ipsilateral eye reduced the neural activity in the visual Wulst elicited by the stimulation of the contralateral eye (Bredenkötter and Bischof 1990a; Michael et al. 2015). This could represent a mechanism of suppression of the information from the non-fixating eye, which may be advantageous for species with mainly monocular vision (Bischof 1988; Rogers 2012).

Although the anatomical asymmetries in the thalamic projections to the visual Wulst are well described, nothing is known on the lateralisation of Wulst neurons’ functional response properties. In chicks hatched from light-exposed eggs, more recrossing projections reach the right visual Wulst from the left thalamus, than those reaching the left visual Wulst from the right thalamus (see Fig. 1a) (Rogers and Sink 1988; Rogers and Deng 1999; Deng and Rogers 2002). This light-dependent anatomical asymmetry clearly has potential implications for the capacity of bilateral information processing of the left and right visual Wulst.

The aim of the present study was: (1) to describe hemispheric asymmetries in the response properties of Wulst visual neurons; (2) to reveal the effect of lateralised embryonic light exposure on the response properties of the visual neurons and on the lateralisation profile. To clarify to which extent chicks’ visual Wulst contributes to bilateral processing, we mainly focused our analysis on the response properties of cells that integrate information from both eyes. We thus conducted extracellular electrophysiological recordings from a large number of visual neurons in the left and right visual Wulst of dark- and light-incubated chicks. The responses of the cells were recorded during stimulation of either the ipsilateral, the contralateral or both eyes with short flashes of light.

## Materials and methods

### Subjects

Thirty-eight laboratory-hatched domestic chicks (*Gallus gallus domesticus*) of the Aviagen ROSS 308 strain were used. Fertilized eggs were obtained from a local commercial hatchery (CRESCENTI Società Agricola S.r.l.–AllevamentoTrepola–cod. Allevamento127BS105/2). Eggs were incubated and hatched within incubators (Marans P140TU-P210TU) at a temperature of 37.7 °C, with 60% humidity in a dark room. After hatching in dark incubators, chicks were isolated and housed individually in metal cages (28 cm wide × 32 cm high × 40 cm deep) with food and water available ad libitum, at a constant room temperature of 30–32 °C and a constant light–dark regime of 14 h light and 10 h dark.

### Experimental treatment

The eggs of the two experimental groups underwent different incubation conditions: ‘Dark Incubated’ group eggs (*N* = 20) were in darkness from embryonic days E0 to E21. ‘Light Incubated’ group eggs (*N* = 18) were light stimulated from the morning of day E18 to the evening of E19 (Fig. 1b). This is the sensitive period to induce anatomical brain lateralisation in chicks (Rogers 1982). During stimulation, light intensity at the level of the eggs was 1036 lx. It was produced by 15 LEDs (270 lm) attached to a rectangular plastic panel (38 × 38 cm^2^) and placed below the roof of the incubator.

### Surgery

On post-hatching day 4, chicks were anesthetized with 0.7 ml of urethane solution (20% urethane in 0.9% NaCl) administered in three to four intramuscular injections with time intervals of 30 min. When a bird became unresponsive to touching and puling of the legs, the head was fixed in a stereotaxic head holder (Bischof 1981) with the beak oriented horizontally. Feathers were removed with wax stripes, the scalp was locally anesthetized with lidocaine gel (2.5% AstraZeneca S.p.A.) and the skull was exposed. Craniotomy was performed above the visual Wulst of both hemispheres and the dura was incised with an injection needle. Both eyelids were fixed by adhesive tape in an open position shortly before the experiment started. The nictitating membrane was kept intact to protect the eye from desiccation during recordings.

### Apparatus and stimuli

The recordings were performed on an anti-vibration table (Thorlab Nexus, 110 × 95 cm) covered with a Faraday cage and placed in a dim light illuminated room. The PsychoPy-tool (v3.0) implemented with Python language was used to create the visual stimuli. A white flashing stimulus was presented for 1 s followed by an inter stimulus interval (ISI) of 4 s. Flashes were presented randomly to the left, the right or both eyes. For each subject, 40 repetitions of each type were presented. Stimuli were presented on a computer monitor (AOC AGON AG271QX; LCD display, size: 27 inches; resolution: 2560 × 1440 pixel Quad HD; refresh rate: 144 Hz; response time: 1 ms) and directed specifically to chick’s eyes by two fibre-optic cables (Carl Zeiss). Exact timing of stimuli presentations was detected by photodiodes attached to the computer monitor outside the lateral walls of the experimental setup (Fig. 1c).

### Neural recording

To record visual units, a 16 channels platinum-iridium microelectrode array (impedance of 2.0 Megohms) was used (MicroProbes for life science, USA). The ground electrode was clamped to the skin of the head. For estimating the brain coordinates for electrode placement, the bregma was used as a 0.0 coordinate. The electrode array (2 × 8 arrangement with a distance of 250 μm between each electrode) covered a region of 1.75 mm in the medial–lateral orientation and 250 μm in the antero-posterior orientation of the brain. It was delimited by the following coordinates: anterior (A) 6.5 mm to A 6.75 mm and lateral (L) 1.0 mm to L 2.75 mm for the right hemisphere or L − 1.0 mm to L − 2.75 mm for the left hemisphere. With a motorized micromanipulator, the electrode array was then slowly lowered into the brain at an angle of 45°. The depth coordinate was *D* 1.3 mm as estimated from the surface of the brain.

Plexon multichannel system (Plexon, Dallas, USA) was used for data acquisition. Signals were pre-amplified with a 16ch head-stage (20 × , Plexon Model number: PX.HST/16V-G20-LN) subsequently amplified 1000 × , digitalised and filtered (300 Hz high-pass filter, 3 kHz low-pass filter and 50 Hz noise removal). Common average referencing (CAR) method (the averaged signal across channels) of the PlexControl system was used for referencing. This provided a good signal-to-noise ratio. Spikes were detected with the PlexControl software with an automatic thresholding set at 4 sigma from the noise level average. The peristimulus time histograms (PSTH) and raster plots of individual units were observed online with the software ‘Neuroexplorer’ (v.5.), which was synchronously running with the PlexControl recording software.

### Histology

**Fig. 2:**
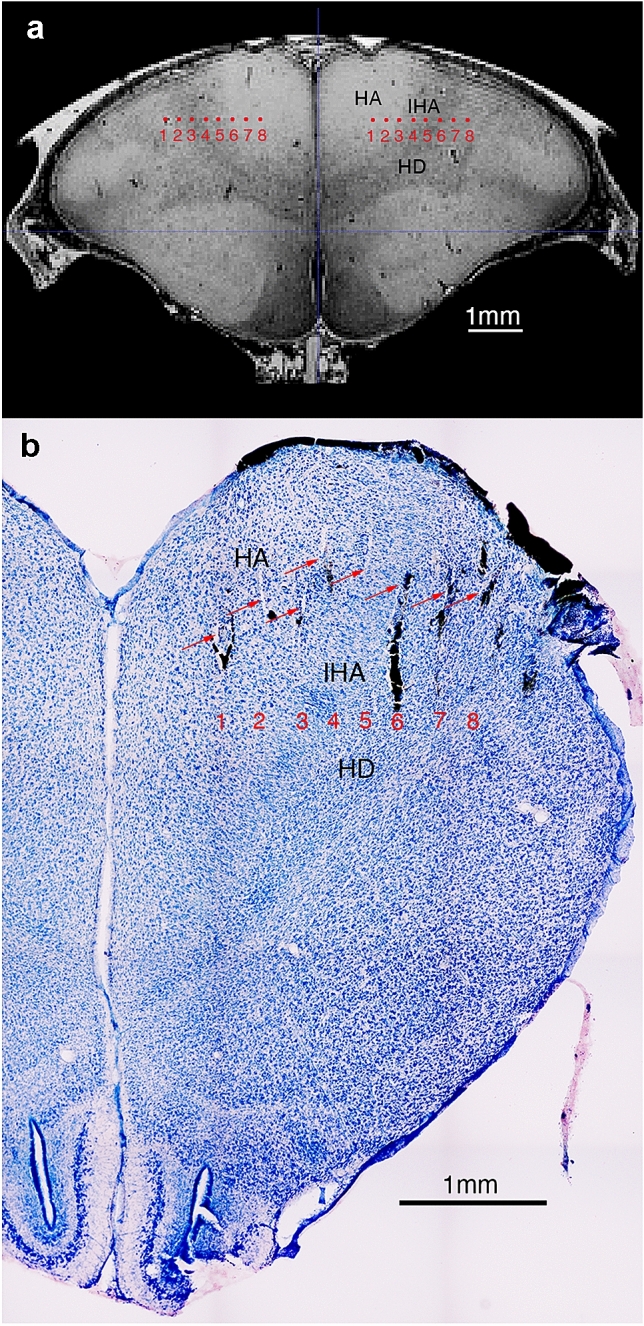
Visualisation of the electrode array placement. **a** Schematic representation of the targeted coordinates in a coronal plane obtained from a MRI scan of a 4-day-old chick (Lorenzi E, Behroozi M, Mayer U, Güntürkün O, Vallortigara G , ‘A three dimensional atlas of the domestic chick brain’, in preparation). Please note that the MRI scan was obtained using the standard 45° head orientation, measured between the beak and ear-bars (as for the electrode placement). Red dots indicate electrode tips (recording site) for the posterior row of the 16 channels (8 × 2) electrode array. Tip position has been estimated based on the stereotactic coordinates used for implantation. **b** Example of visible electrode tracks, in histological coronal brain sections of the right hemisphere. Red arrows indicate visible tracks, while the red numbers (1–8) indicate the expected position of electrode tips, based on the deepest electrode track visible (number 6 in this case). *HA* hyperpallium apicale, *IHA* nucleus interstitialis hyperpallii apicale, *HD* hyperpallium densocellulare

At the end of each experiment, chicks were overdosed with ketamine/xylazine solution (1:1 ketamine 10 mg/ml + xylazine 2 mg/ml) and perfused with phosphate-buffered saline (PBS; 0.1 mol, pH = 7.4, 0.9% sodium chloride, 5 °C) and 4% paraformaldehyde (PFA). Brains were incubated for at least 2 days in PFA containing 20% sucrose and further 2 days in 30% Sucrose in PFA. Coronal 60 μm sections were cut at − 20 °C using a cryostat (Leica CM1850 UV), mounted on glass slides, stained with Giemsa dye (MG500, Sigma-Aldrich, St. Louis, USA) and cover slipped with Eukitt (FLUKA). Brain’s sections were examined with a Zeiss stereomicroscope (Stemi 508, Carl Zeiss, Oberkochen, Germany). The target position and an example of visible electrode tracks in an histological brain section are shown in Fig. 2.

### Data analysis

Spikes were sorted with the PlexonOfline Sorter software (v3.3.5., Plexon Inc). Principal component clusters were automatically sorted with the ‘K-Means scan’ method. The data were then processed with Matlab (R2018a). All further analyses were based on the PSTH (bin size 50 ms) of each individual unit. Only strongly visually responsive units were taken for the analysis. For this purpose, the peak firing rate during the 1 s of stimulus presentation (ON response) or during the 1 s after stimulus presentation (OFF response) had to be twofold higher than any peak occurring in the 1 s before stimulus presentation. This criterion had to be fulfilled in at least one of the presentation conditions (ipsilateral, contralateral and bilateral presentation).

**Fig. 3:**
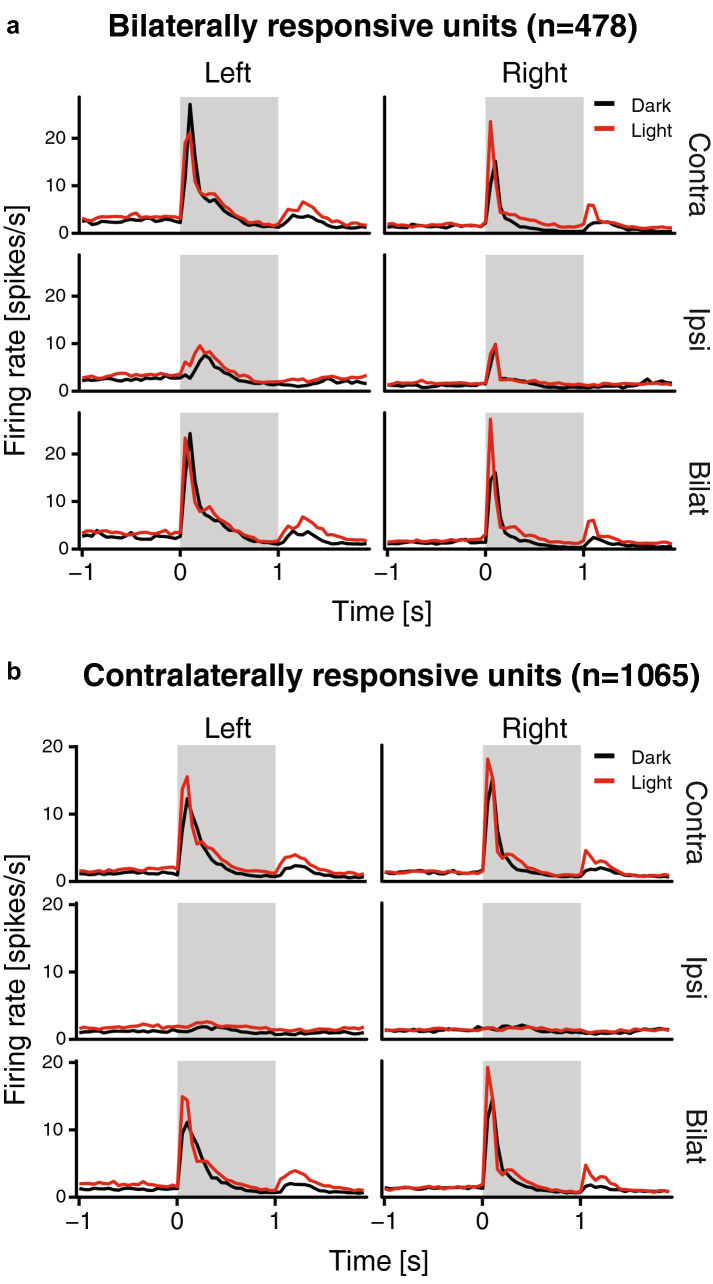
Average peristimulus time histograms, showing the responses of bilaterally responsive units (**a**) and contralaterally responsive units (**b**) to the contralateral, ipsilateral or bilateral stimulation. Data from units recorded in the left and right visual Wulst are shown separately side by side. Black and red lines represent units recorded from dark- and light-incubated animals, respectively

Bilaterally responsive cells were then extracted from the overall population of visually responsive units, based on the selection steps described in the following paragraph. The aim was to select all units that showed any indication of bilateral integration and to separate them from the units that showed almost exclusively response to contralateral eye stimulation (Fig. 3). At each step, we selected out from the overall sample all the units with a given feature indicative of bilateral integration. Thus, to be considered as bilaterally responsive, each unit had to satisfy at least one of the criteria. At each step units with the peak firing rate lower than 5 Hz were excluded from further analyses. All selection steps were applied to both ON responses (during 1 s stimulus presentation) and OFF responses (during 1 s after stimulus presentation).

Since virtually all cells showed responses to contralateral stimulation, the first indication of bilateral integration was the presence of an additional response to ipsilateral eye stimulation. In this step, we thus selected units whose ipsilateral peak response was at least 4 sigma (standard deviations, SD) higher than the average noise level. One cell responded only to ipsilateral stimulation and was thus excluded from any further analysis.

Bilateral integration can be indicated also by modulation of the contralateral response in the presence of concurrent ipsilateral stimulation. Thus, in the second step, from the remaining population we selected units whose contralateral firing rate peak was significantly higher or lower than the bilateral one. This was done by Wilcoxon rank sum test with continuity correction (*p* < 0.05) comparing the peaks of the contralateral and bilateral responses.

In the third step, we compared the responses occurring during the whole 1 s of stimulus presentation (or 1 s after stimulus presentation) in the contralateral and bilateral stimulation condition, since bilateral integration can occur also outside the peak response. This was done by Wilcoxon rank sum test with continuity correction (*p* < 0.05) using the 20 bins (50 ms each), as data points.

At the end, the different bilaterally responsive units extracted through each selection step were pooled together creating the group that included all the cells showing any sign of bilateral integration, whereas the rest of the units composed the population of contralaterally responsive cells. All further statistical analyses were run separately for each of these two populations.

### Statistics

All statistical analysis was performed using R (R Core Team, 2020, with the packages: “rcompanion”; “FSA”; “ggplot2”; “tidyverse”). Non-parametric statistical approaches were used for all analyses. The dependent variables that we analysed were: the firing rate in the 1 s before stimulus onset, averaged over all the stimulation conditions (spontaneous firing rate); the firing rate peak occurring during the 1 s of stimulus presentation (ON peak); the latency of the ON peak (measured as the middle of the bin in which the peak occurred); the firing rate peak occurring during the 1 s after the end of the stimulus presentation (OFF peak); the latency of the OFF peak. For the analyses we subtracted the spontaneous firing rate from the firing rate peaks.

For each dependent variable, we analysed the effect of “treatment” (light incubation) and “hemisphere” as well as an interaction between these factors, using Scheirer–Ray–Hare test. Significant interactions of the two factors were further analysed post hoc with Dunn’s Kruskal–Wallis test for multiple comparisons (Dunn 1964).

## Results

We successfully isolated 1544 visually responsive units. Of these, 478 units (Fig. 3a) were responsive to stimulation of both eyes (see Methods for selection criteria), one unit was responsive to only ipsilateral response (excluded from further analysis), while the remaining 1065 were almost exclusively responsive to contralateral stimulation (Fig. 3b). Already by visual observation of the average PSTH’s of all units of the two different populations, several instances of both light-dependent and light-independent lateralisation could be detected. The light treatment seemed to impact the spontaneous firing rates before stimuli onsets as well as the firing rate peak and latency of the ON and OFF responses. These effects were systematically analysed step by step in each of the two populations.

### Results for bilaterally responsive cells

**Fig. 4:**
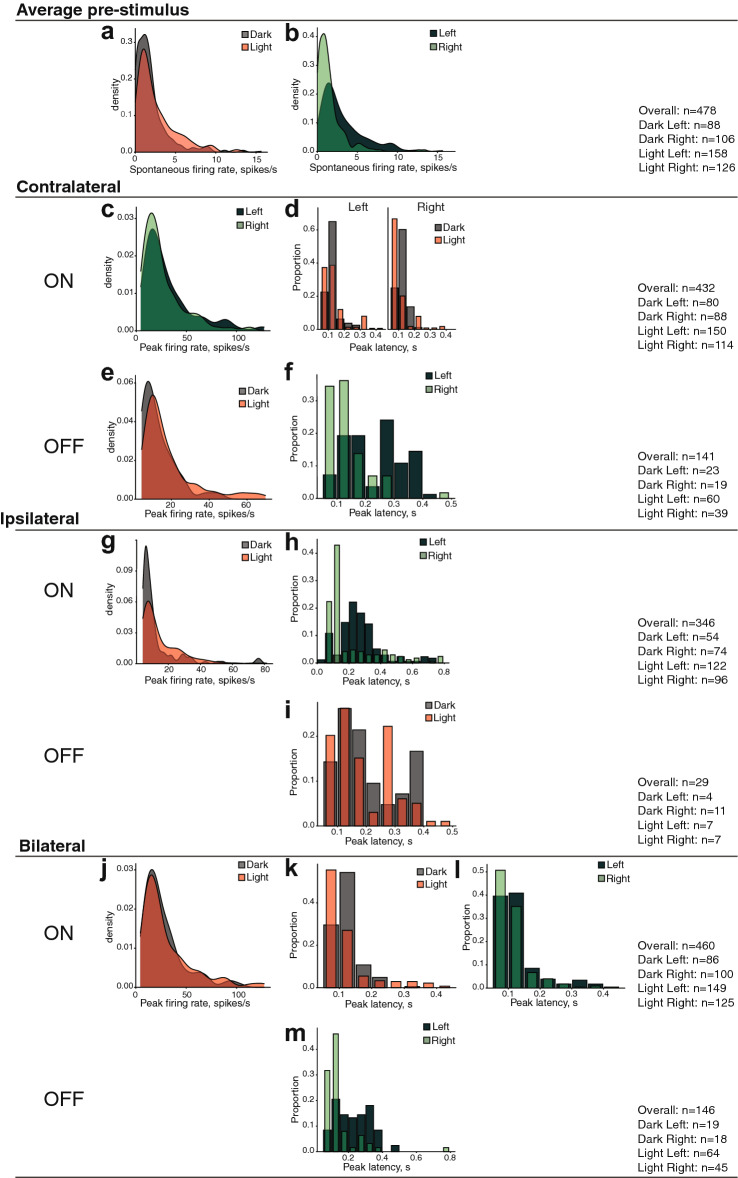
Graphs illustrating all the statistically significant effects emerging for the bilaterally responsive units. **a** Kernel density plot showing the distribution of units with various spontaneous firing rates for the light-incubated (red) and dark-incubated (grey) conditions, collapsed for the two hemispheres. On the *y*-axis, the density of units (in proportion to the overall recorded sample), on the *x*-axis the firing rate (in spikes per second). For instance, in this graph it can be seen that more units with low firing rate were present in the dark group (the grey curve is higher than the red curve at lower firing values), while higher spontaneous firing rates were present in the light-incubated group (the red curve is above the grey one at higher firing rates). **b** Density plot for the spontaneous firing rates in the left (darker green) and right (lighter green) hemispheres. The graphs at **c**, **e**, **g**, **j** show density plots for the peak firing rate of the contralateral ON, contralateral OFF, ipsilateral ON and ipsilateral OFF responses, respectively. The graphs at **d**, **f**, **h**, **i**, **k**, **l**, **m** show the proportion of units with different peak latencies. Peak latency is shown on the *y*-axis, while the proportion of units (on the total recorded sample) is on the *y*-axis. Data from the light- and dark-incubated conditions are represented by the red and grey columns, while data from the left and right hemispheres are shown in darker and lighter green respectively. In **d**, data from the left and right hemispheres are presented separately side by side. In this graph, for instance, it can be seen that the right hemisphere of the light-incubated group contains more neurons with shorter peak latencies than that of the dark-incubated one

Statistical analyses revealed several significant effects. All results are summarised in Table 1, while Fig. 4 further illustrates the significant effects.

**Table 1:** Summary of the results of the statistical analyses for bilaterally responsive cells

	Main results (Scheirer–Ray–Hare)		
	Treatment	Hemisphere	Interaction (Treat × Hemi)
Average pre-stimulus			
Spontaneous firing rate	*H*_(1)_ = 11.7; ***p * = 0.001**	*H*_(1)_ = 63.123; ***p *** **< 0.001**	*H*_(1)_ = 0.006; *p* = 0.938
Contralateral			
ON: peak firing rate	*H*_(1)_ = 0.121; *p* = 0.728	*H*_(1)_ = 5.389; ***p *** **= 0.02**	*H*_(1)_ = 1.91; *p* = 0.167
ON: peak latency	*H*_(1)_ = 10.606; ***p *** **= 0.001**	*H*_(1)_ = 13.754; ***p *** **< 0.001**	*H*_(1)_ = 7.452; ***p *** = 0.006*
OFF: peak firing rate	*H*_(1)_ = 6.037; ***p *** **= 0.014**	*H*_(1)_ = 0.002; *p* = 0.966	*H*_(1)_ = 1.705; *p* = 0.192
OFF: peak latency	*H*_(1)_ = 0.5; *p* = 0.48	*H*_(1)_ = 34.6; ***p *** **< 0.001**	*H*_(1)_ = 0.041; *p* = 0.84
Ipsilateral			
ON: peak firing rate	*H*_(1)_ = 8.769; ***p *** **= 0.003**	*H*_(1)_ = 0.007; *p* = 0.938	*H*_(1)_ = 0.954; *p* = 0.329
ON: peak latency	*H*_(1)_ = 3.2; *p* = 0.074	*H*_(1)_ = 36.707; ***p *** **< 0.001**	*H*_(1)_ = 2.402; *p* = 0.121
OFF: peak firing rate	*H*_(1)_ = 0.093; *p* = 0.76	*H*_(1)_ = 0.028; *p* = 0.866	*H*_(1)_ = 1.811; *p* = 0.178
OFF: peak latency	*H*_(1)_ = 4.428; ***p *** **= 0.035**	*H*_(1)_ = 0.296; *p* = 0.586	*H*_(1)_ = 0.011; *p* = 0.916
Bilateral			
ON: peak firing rate	*H*_(1)_ = 4.101; ***p *** **= 0.043**	*H*_(1)_ = 0.793; *p* = 0.373	*H*_(1)_ = 3.335; *p* = 0.068
ON: peak latency	*H*_(1)_ = 14.539; ***p *** **< 0.001**	*H*_(1)_ = 7.897; ***p *** **= 0.005**	*H*_(1)_ = 2.411; *p* = 0.120
OFF: peak firing rate	*H*_(1)_ = 2.458; *p* = 0.117	*H*_(1)_ = 0.109; *p* = 0.741	*H*_(1)_ = 1.933; *p* = 0.164
OFF: peak latency	*H*_(1)_ = 0.618; *p* = 0.432	*H*_(1)_ = 31.434; ***p *** **< 0.001**	*H*_(1)_ = 0.251; *p* = 0.616
*Post hoc (Dunn 1964)			
Dark: left vs. right	Light: left vs. right	Left: dark vs. light	Right: dark vs. light
*z* = 0.1972; *p* = 0.843	*z* = 4.600; ***p *** **< 0.001**^a^	*z* = 0.6537; *p* = 0.513	*z* = 4.4517; ***p *** **< 0.001**^a^

^a^Significant also after a Bonferroni adjustment for multiple comparisons

*Significant interaction, for which an additional post hoc (Dunn 1964) analysis has been performed (reported at the bottom of the table). Significant results (*p* < 0.05) are in bold

The spontaneous firing rate in the pre-stimulus phase was analysed collapsing the data of all the ipsilateral, contralateral and bilateral stimulation conditions (this variable refers to the time window before the stimulation occurs; see Fig. 4 for the number of units in each condition). The spontaneous firing rate was significantly different between the light- and the dark-incubated condition, as well as between the left and right hemispheres, with no significant interaction between the two factors. As regards the effect of light, more neurons with higher spontaneous firing rate were present in the light-incubated group, regardless of the hemisphere (Fig. 4a). As regards the hemispheric asymmetry, more neurons with higher spontaneous firing rate were present in the left hemisphere, regardless of the incubation condition (Fig. 4b).

For the analysis of the contralateral ON responses, we selected only units whose ON response was at least 4 SD above the average firing rate. The firing rate peak (normalised by subtracting the spontaneous firing rate) was significantly different between the two hemispheres (more units with a higher response peak were present in the left hemisphere, Fig. 4c). Light had no significant effect, nor did it interact with the lateralisation effect of the response peak firing rate. However, light-incubation had a strong lateralisation effect on the latency of the ON responses as revealed by the significant “treatment” × “hemisphere” interaction for this dependent variable. The post hoc analysis of this effect (reported at the bottom of Table 1) revealed that in the right hemisphere only, light incubation caused the presence of more units with faster responses compared to dark incubation. Moreover, in the light-condition only, more neurons with shorter latency were found in the right than in the left hemisphere. For the dark-incubated group, no lateralisation effect was present in the latency of the contralateral ON responses (Fig. 4d).

For the analysis of the contralateral OFF responses, we selected only units whose OFF response was at least 4 SD above the average firing rate. Contralateral OFF responses were also affected by in-egg light exposure: more units with higher OFF response peaks were present in the light-incubation condition, regardless of the hemisphere (Fig. 4e). Moreover, the latency of the OFF response peak was shorter in the right hemisphere, regardless of the treatment condition (light or dark incubation) (Fig. 4f).

For the ipsilateral ON responses, a higher firing rate peak was more often present in the light-incubation condition (regardless of the hemisphere) (Fig. 4g). Moreover, regardless of the treatment condition, more neurons with faster responses were present in the right hemisphere (Fig. 4h).

For the ipsilateral OFF responses, the only significant effect emerged comparing the latency of the peak response in the two incubation conditions. A wider range or response latencies appeared to be present in the light-incubated group (Fig. 4i).

For the bilateral ON responses, more units with higher firing rate peak (Fig. 4j) and shorter latencies (Fig. 4k) were present in the light-incubation condition, regardless of the hemisphere. Moreover, a higher proportion of units with shorter latencies could be observed in the right hemisphere (Fig. 4l), regardless of the incubation condition.

For the bilateral OFF responses, more neurons with shorter latencies were present in the right hemisphere (Fig. 4m), irrespective of the treatment condition.

**Fig. 5:**
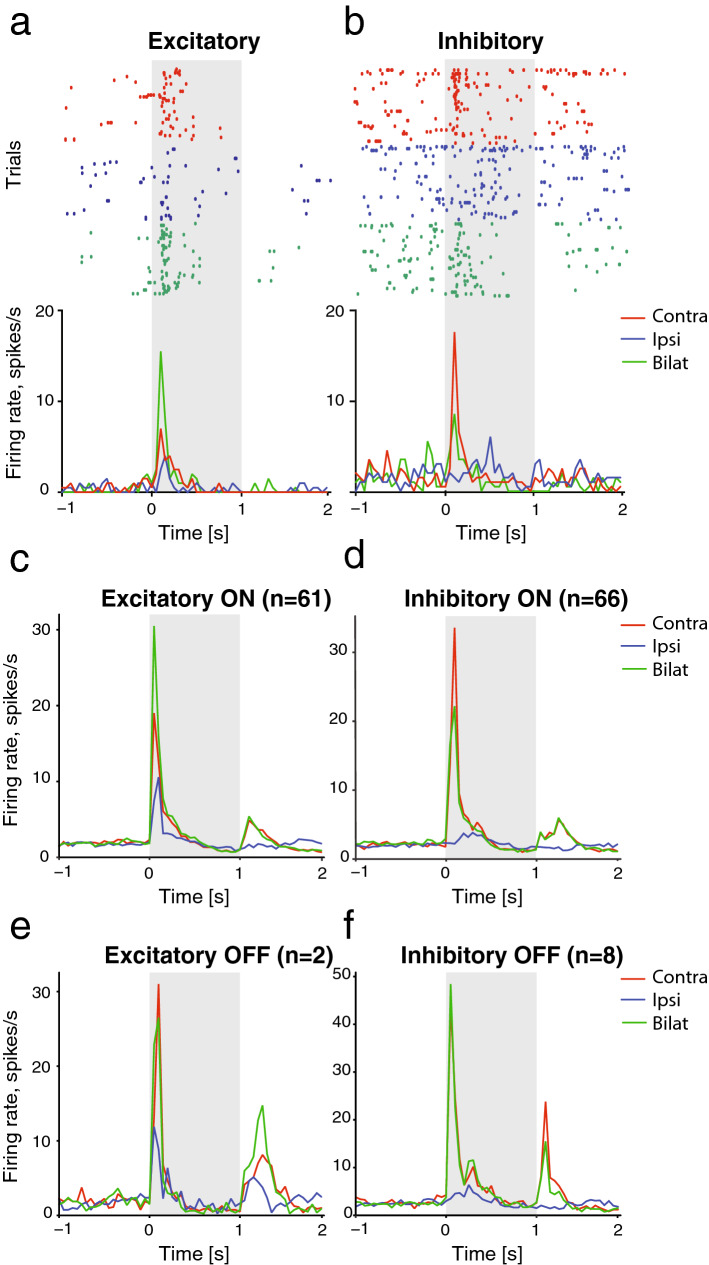
Excitatory and inhibitory responses to simultaneous stimulation of both eyes. Two examples of a unit showing an excitatory (**a**) and an inhibitory (**b**) response to bilateral stimulation. Raster plots with peristimulus time histograms (PSTHs) are shown, with responses to contralateral stimulation depicted in red, responses to ipsilateral stimulation in blue and to bilateral in green. The PSTHs at **c**–**f** represent the average responses of all the excitatory ON, inhibitory ON, excitatory OFF and inhibitory OFF units, respectively

Furthermore, we analysed the presence of excitatory and inhibitory interactions caused by the simultaneous stimulation of the two eyes. We considered a unit to show an excitatory bilateral response, if the response to the bilateral stimulation was significantly higher compared to the response to contralateral stimulation (as revealed by a Wilcoxon rank sum test with continuity correction, *p* < 0.05). Likewise, a response was considered inhibitory, when the bilateral stimulation elicited significantly lower response than the contralateral one. Examples of two units with excitatory and inhibitory responses to bilateral stimulation are shown in Fig. 5a, b, respectively. Overall, we were able to isolate 61 ON units showing excitatory integration (Fig. 5c) and 66 ON units with inhibitory integration (Fig. 5d), out of 432 units with a contralateral ON response. Moreover, by focusing only on the OFF responses (regardless of the potential presence of ON responses from the same cells), we isolated 2 OFF units with an excitatory integration (Fig. 5e) and only 8 OFF units with an inhibitory integration (Fig. 5f), out of 141 units with a contralateral OFF response.

### Results for contralaterally responsive cells

**Fig. 6:**
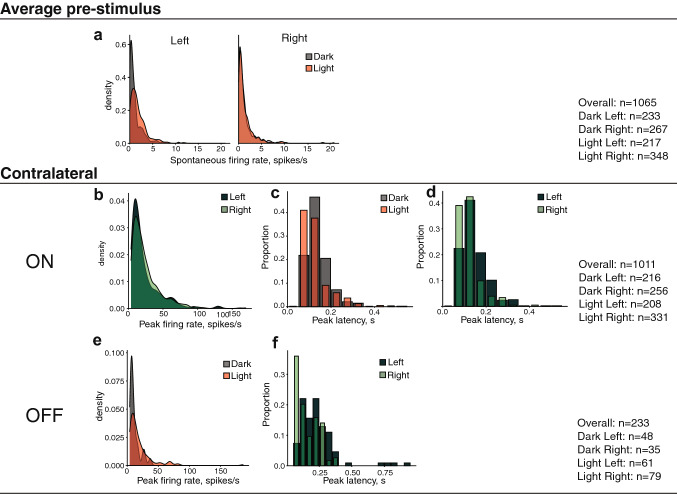
Graphs illustrating all the statistically significant effects emerging for the contralaterally responsive units. **a** Density plots for the spontaneous firing rates, presented separately for the left and right hemispheres. Graphs at **b**, **e** show density plots for the peak firing rate of the contralateral ON and OFF responses, respectively. The remaining graphs show the proportion of units with different peak latencies for the contralateral ON responses (**c**, **d**) and the contralateral OFF responses (**f**)

In case of purely contralaterally responsive cells too, several significant effects emerged, which are summarised in Table 2, while Fig. 6 further illustrates the significant effects.

**Table 2:** Summary of the results of the statistical analyses for contralaterally responsive cells

	Main results (Scheirer–Ray–Hare)		
	Treatment	Hemisphere	Interaction (Treat × Hemi)
Average pre-stimulus			
Spontaneous firing rate	*H*_(1)_ = 15.845; ***p *** **< 0.001**	*H*_(1)_ = 9.653; ***p *** **= 0.002**	*H*_(1)_ = 12.414; p < 0.001*
Contralateral			
ON: peak firing rate	*H*_(1)_ = 1.42; *p* = 0.233	*H*_(1)_ = 5.119; ***p *** **= 0.024**	*H*_(1)_ = 1.468; *p* = 0.226
ON: peak latency	*H*_(1)_ = 33.02; ***p *** ** < 0.001**	*H*_(1)_ = 43.846; ***p *** **< 0.001**	*H*_(1)_ = 0.709; *p* = 0.4
OFF: peak firing rate	*H*_(1)_ = 13.854; ***p *** **< 0.001**	*H*_(1)_ = 0.333; *p* = 0.564	*H*_(1)_ = 2.027; *p* = 0.155
OFF: peak latency	*H*_(1)_ = 0.666; *p* = 0.415	*H*_(1)_ = 22.394; ***p *** **< 0.001**	*H*_(1)_ = 0.9; *p* = 0.343
*Post hoc (Dunn 1964)			
Dark: left vs. right	Light: left vs. right	Left: dark vs. light	Right: dark vs. light
*z* = − 0.3783; *p* = 0.705	*z* = 4.682; ***p *** **< 0.001**^a^	*z* = − 5.426; ***p *** **< 0.001**^a^	*z* = − 0.897; *p* = 0.369

^a^Significant also after a Bonferroni adjustment for multiple comparisons

*Significant interaction, for which an additional post hoc (Dunn 1964) analysis has been performed (reported at the bottom of the table). Significant results (*p* < 0.05) are in bold

Contrary to what was observed for bilaterally responsive units, the spontaneous firing rate (average pre-stimulus in Table 2) showed a significant interaction of the factors “incubation treatment” and “hemisphere”. This indicated that the effect of light was lateralised. Light-incubation had a strong effect only in the left hemisphere, where neurons showed higher spontaneous firing rate compared to the dark-incubation condition. This produced also a significant leftward lateralisation of the spontaneous firing rate, within the light-incubated condition (see the bottom of Table 2 for the post hoc analysis). On the contrary, the dark-incubated condition did not reveal any asymmetry (Fig. 6a).

The analysis of the contralateral ON firing rate peak revealed significant lateralisation, with more units showing a higher firing rate in the right hemisphere (Fig. 6b). Light had no effect on the peak firing rate, nor did it interact with this lateralisation effect. However, the latency of the response peak was affected by light incubation, with more units showing a shorter latency in the light-incubated group, regardless of the hemisphere (Fig. 6c). Moreover, there was also a light-independent lateralisation effect, with faster responses in the right hemisphere, regardless of the incubation condition (Fig. 6d).

Finally, in the light-incubated group, we observed more cells with stronger contralateral OFF responses, regardless of the hemisphere (Fig. 6e). Moreover, shorter peak latencies were more often present in the right hemisphere, regardless of the treatment (Fig. 6f).

## Discussion

The aims of the current paper were to investigate the presence of asymmetries in the visual response properties in the left and right visual Wulst and, most importantly, to test the impact of embryonic light stimulation on the response properties, in general, and on their lateralisation profile.

Several instances of lateralisation emerged both in the population of bilaterally responsive units and among contralaterally responsive units. Most of these forms of lateralisation, however, appeared independently from the light-incubation condition. We found only two instances of light-dependent lateralisation. In bilateral cells, responses to the onset of a contralateral stimulus (contralateral ON responses) were lateralised only in the light-incubated group. The dark-incubated group showed no differences between the two hemispheres and was characterised by slower responses. The right hemisphere of the light-incubated group showed faster responses compared to that of the dark-incubated group and compared to the left hemisphere of the light condition (Fig. 4d).

The presence of light-dependent lateralisation in bilaterally responsive units of the right visual Wulst is, at least partially, in line with previous anatomical studies (e.g., Rogers and Deng 1999). Indeed, these works showed increased re-crossing projections from the left OPT to the right visual Wulst after embryonic light stimulation (Fig. 1a). On this basis, we expected the right visual Wulst to be the one prevalently affected by light exposure. However, we expected to find significant light-dependent lateralisation in the responses to bilateral and/or ipsilateral stimulation, since this information would be transferred through the stronger recrossing connection to the right Wulst of the light-incubated group. Although, for bilateral stimulation, a non-significant trend emerged for light-dependent lateralisation in the peak firing rate of the ON responses (*p* = 0.068), no other measures approached statistical significance (Table 1). On the contrary, what we found was significant light-dependent lateralisation in the right Wulst’s responses to contralateral stimulation (left eye). This surprising result suggests the presence of further forms of underlying anatomical lateralisation, in addition to what was revealed by the existing neural tracing studies (Rogers and Sink 1988; Rogers and Bolden 1991; Rajendra and Rogers 1993; Rogers and Deng 1999).

It is also noteworthy that the original tracing studies do not clarify whether the recrossing projections that are strengthened by light exposure are excitatory or inhibitory. It has been theorised that a potential function of bilateral information integration in birds with laterally placed eyes could be the inhibition of competing information from the non-fixating eye (Bischof 1988; Rogers 2012).

In line with that, ipsilateral stimulation has been reported to inhibit the overall activity of the visual Wulst in zebra finches. This is revealed by reduced activity during bilateral stimulation, compared to contralateral stimulation only (Michael et al. 2015). In our recordings, however, we were able to isolate two equally large subpopulations of units showing either excitatory or inhibitory responses to the synchronous stimulation of the two eyes. It is clear that we are just beginning to understand the functions of bilateral integration in the visual Wulst in birds with laterally placed eyes. Future studies could thus be devoted to clarifying the role of inhibitory and excitatory networks in the development of light-dependent lateralisation.

The second instance of light-dependent lateralisation was found in the purely contralaterally responsive cells. In this case, light exposure determined a selective increase of the spontaneous firing rate of the left hemisphere, causing a lateralisation, which was not present in the dark-incubated condition. Thus, in addition to the known asymmetry of the recrossing supraoptic decussation to the right Wulst (Rogers and Deng 1999), light exposure induces other forms of lateralisation affecting directly the left visual Wulst. Light exposure during embryonic development stimulates the right eye, whose major projections are to the left visual Wulst. As a consequence, those neurons in the left visual Wulst that are responsive to contralateral stimulation are increasingly activated during in-egg development. We found that this has an impact on the physiology, which persists also after hatching, as reflected by the increase in the spontaneous firing rate of this population. Our results thus extend the original findings by Rogers and collaborators (Rogers and Sink 1988; Rogers and Bolden 1991; Rajendra and Rogers 1993; Rogers and Deng 1999), showing for the first time the presence of light-induced lateralisation in the physiological response properties of the left visual Wulst. This asymmetry may reflect changes that are not detectable at the level of neural projections, and thus may have escaped previous tracing studies. Future investigations should seek to identify the cellular and morphological mechanisms underlying this functional asymmetry. This can be done by investigating light-induced changes in the morphology, density and connectivity of specific neural populations in the left visual Wulst.

Intriguingly, we found that in bilaterally responsive units, embryonic stimulation of the right eye had an impact on the visual Wulst of both hemispheres. Bilaterally responsive cells, by definition receive also ipsilateral information. Thus, stimulation of the right eye by in-egg light exposure, reaches both the left and also the right visual Wulst (through the recrossing projections). This explains why the spontaneous firing rate of bilaterally responsive cells is increased by light-incubation in both hemispheres and does not show light-induced asymmetries.

Moreover, we found ample evidence of a hemisphere-independent effect of light on the maturation of visually responsive neurons in both bilaterally and contralaterally responsive units. Exposure to light increased the overall baseline activity in bilaterally responsive units (Fig. 3a), as revealed by the spontaneous firing rate. Light exposure also increased the diversity of response properties (e.g., the range of firing rate peaks and latencies displayed by the units, see Figs. 3e, g, i–k, 5c–e). This implied often an increase of the peak firing rate and faster responses. The effect of light appeared to be particularly pronounced for the units with OFF responses (i.e., responses to the disappearance of the visual stimulus, see Fig. 2). We believe that light exposure increased the sensitivity of these units to changes in the visual stimulation. These general changes of visual response properties show that in-egg light exposure not only induces asymmetries at the neural or behavioural level, but also affects the maturation of the visual system. Until now, studies on in-egg light exposure focused mostly on its effects on lateralisation. For instance, behavioural differences between light- and dark-incubated chicks were interpreted as uniquely deriving from the different lateralisation profiles of these two groups (e.g., Rogers 2000; Daisley et al. 2010). While these lateralisation effects and their influence on cognitive performance is undeniable, future studies should pay increased attention to the potential general effect of light-exposure on the maturation of the visual system. It would be particularly important to confirm that the hemisphere-independent physiological changes, which we found, are indeed reflected in the behavioural performance of the animals. This could be done focusing on the presence of behavioural changes, occurring regardless of the eye-system used by the animals, in basic visual functions (e.g., visual acuity, detection times, pattern discrimination).

Another important finding of the current study was the presence of multiple light-independent lateralisation effects in the visual Wulst. This provides the first direct evidence that lateralisation in neural response properties can occur in the absence of asymmetric light stimulation. Multiple instances of light-independent lateralisation have already been reported at the behavioural level (e.g., Vallortigara et al. 2001; Mascetti and Vallortigara 2001; Deng and Rogers 2002; Chiandetti 2011; Chiandetti et al. 2013, 2017; Chiandetti and Vallortigara 2019). However, until recently, only few studies investigated the presence of similar effects at the neurobiological level (e.g., Johnston et al. 1995). In our recent works, we started to tackle this issue reporting cases of neurobiological lateralisation in various brain areas of dark-incubated chicks (e.g., Mayer et al. 2017; Lorenzi et al. 2019; Morandi-Raikova and Mayer 2020; Morandi-Raikova et al. 2021; see also Mayer et al. 2016; Lorenzi et al. 2017; 2019; Golüke et al. 2019; Corrales Parada et al. 2021 for similar trends). Intriguingly, in our latest work (Morandi-Raikova et al. 2021), we described a case anatomical lateralisation in the entopallium of dark-incubated chicks. In this study, higher density of parvalbumin-expressing neurons, which are likely to represent a sub-class of GABAergic inhibitory cells, was found in the right entopallium. The entopallium is the higher processing station of the tectofugal visual pathway (Karten and Shimizu 1989; Johnston and Colombo 2017). Species such as pigeons have long been known to present lateralisation in the tectofugal pathway (Güntürkün 1997; Güntürkün et al. 1998; Manns and Güntürkün 1999a, b, 2003; Keysers et al. 2000; Skiba et al. 2002; Folta et al. 2004; Verhaal et al. 2012; Ströckens et al. 2013; Freund et al. 2016; Stacho et al. 2016; Güntürkün and Ocklenburg 2017), but not in the thalamofugal one. On the contrary, in domestic chicks lateralisation was considered to be mostly confined to the thalamofugal visual pathway (e.g., Rogers and Deng 1999), creating a dissociation between the two species. In any case, the classical view was that in both species, anatomical lateralisation of the visual system derived almost exclusively from light exposure (e.g., see Letzner et al. 2020 for recent research in pigeons). The most recent literature, including the current work, is now starting to paint a more complex picture. We now know that, even though differences between species are undeniable, both model species present lateralisation of the tectofugal visual pathway (while thalamofugal lateralisation in pigeons has still not been reported). Moreover, in the current study, while we confirm that light exposure determines some asymmetries, we also describe abundant cases of light-independent lateralisation in the response properties of the main thalamofugal end station.

One interesting aspect of these light-independent lateralisation effects is the frequent occurrence of shorter response latencies in the right visual Wulst (see Figs. 3f, h, l, m, 5d, f). This is in line with behavioural studies showing specialization of the left eye-system (right hemisphere) for “ready response to releaser” functions (Andrew 2009). While the left hemisphere shows a superior ability to sustain an initiated response to visual stimuli, the right hemisphere plays a dominant role in enacting primitive wariness and avoidance reactions. This is based on its specialization to fast responses to releasers of species-specific behaviours and to novel stimuli. This supports, for instance, the specialization of the left eye-system for monitoring biologically relevant stimuli, like predators and conspecifics (e.g., see Rogers 2000; Dharmaretnam and Rogers 2005). The faster responses to visual stimulation that we see in the right visual Wulst may be part of a mechanism to support these specializations.

In conclusion, we find that embryonic light stimulation plays an important role for the development of the visual system, going beyond its contribution to the emergence of some forms of lateralisation. Moreover, we provide the first direct evidence of lateralisation in the neural response properties of units of the visual Wulst, both in a light-dependent and light-independent fashion. The presence of lateralisation independent from light-exposure, which was a very frequent finding in our results, is of particular importance for our understanding of the mechanisms behind lateralized brain development.

## Data Availability

The data that support the findings of this study are available from the corresponding author upon reasonable request.

## Code availability

The custom codes that were used for data analysis are available from the corresponding author upon reasonable request.

## References

[CR1] Andrew RJ (1991). Neural and behavioural plasticity: the use of the domestic chick as a model.

[CR2] Andrew RJ (2009). Origins of asymmetry in the CNS. Semin Cell Dev Biol.

[CR3] Andrew RJ, Johnston ANB, Robins A, Rogers LJ (2004). Light experience and the development of behavioural lateralisation in chicks II. Choice of familiar versus unfamiliar model social partner. Behav Brain Res.

[CR4] Atoji Y, Sarkar S, Wild JM (2018). Differential projections of the densocellular and intermediate parts of the hyperpallium in the pigeon (*Columba livia*). J Comp Neurol.

[CR5] Bischof H-J (1981). A stereotaxic headholder for small birds. Brain Res Bull.

[CR6] Bischof H-J (1988). The visual field and visually guided behavior in the zebra finch (*Taeniopygia guttata*). J Comp Physiol.

[CR7] Bischof H-J, Watanabe S (1997). On the structure and function of the tectofugal visual pathway in laterally eyed birds. Eur J Morphol.

[CR8] Bischof H-J, Eckmeier D, Keary N (2016). Multiple visual field representations in the visual Wulst of a laterally eyed bird, the zebra finch (*Taeniopygia guttata*). PLoS ONE.

[CR9] Blake R, Wilson H (2011). Binocular vision. Vis Res.

[CR10] Bredenkötter M, Bischof H-J (1990). Differences between ipsilaterally and contralaterally evoked potentials in the visual Wulst of the zebra finch. Vis Neurosci.

[CR11] Bredenkötter M, Bischof H-J (1990). Ipsilaterally evoked responses of the zebra finch visual Wulst are reduced during ontogeny. Brain Res.

[CR12] Budzynski CA, Gagliardo A, Ioalé P, Bingman VP (2002). Participation of the homing pigeon thalamofugal visual pathway in sun-compass associative learning. Eur J Neurosci.

[CR13] Buschmann J-UF, Manns M, Güntürkün O (2006). “Let There be Light!” Pigeon eggs are regularly exposed to light during breeding. Behav Process.

[CR14] Chiandetti C (2011). Pseudoneglect and embryonic light stimulation in the avian brain. Behav Neurosci.

[CR15] Chiandetti C, Vallortigara G (2019). Distinct effect of early and late embryonic light-stimulation on chicks’ lateralization. Neuroscience.

[CR16] Chiandetti C, Galliussi J, Andrew RJ, Vallortigara G (2013). Early-light embryonic stimulation suggests a second route, via gene activation, to cerebral lateralization in vertebrates. Sci Rep.

[CR17] Chiandetti C, Lemaire B, Versace E, Vallortigara G (2017). Early- and late-light embryonic stimulation modulates similarly chicks’ ability to filter out distractors. Symmetry.

[CR18] Clark WJ, Colombo M (2020). The functional architecture, receptive field characteristics, and representation of objects in the visual network of the pigeon brain. Prog Neurobiol.

[CR19] Corrales Parada CD, Morandi-Raikova A, Rosa-Salva O, Mayer U (2021). Neural basis of unfamiliar conspecific recognition in domestic chicks (*Gallus Gallus domesticus*). Behav Brain Res.

[CR20] Cowan WM, Adamson L, Powell TPS (1961). An experimental study of the avian visual system. J Anat.

[CR21] Daisley JN, Vallortigara G, Regolin L (2010). Logic in an asymmetrical (social) brain: transitive inference in the young domestic chick. Soc Neurosci.

[CR22] Deng C, Rogers LJ (1997). Differential contributions of the two visual pathways to functional lateralization in chicks. Behav Brain Res.

[CR23] Deng C, Rogers LJ (2002). Social recognition and approach in the chick: lateralization and effect of visual experience. Anim Behav.

[CR24] Denton CJ (1981). Topography of the hyperstriatal visual projection area in the young domestic chicken. Exp Neurol.

[CR25] Dharmaretnam M, Rogers LJ (2005). Hemispheric specialization and dual processing in strongly versus weakly lateralized chicks. Behav Brain Res.

[CR26] Dunn OJ (1964). Multiple comparisons using rank sums. Technometrics.

[CR27] Folta K, Diekamp B, Güntürkün O (2004). Asymmetrical modes of visual bottom-up and top-down integration in the thalamic nucleus rotundus of pigeons. J Neurosci.

[CR28] Frasnelli E, Vallortigara G, Rogers LJ (2012). Left–right asymmetries of behaviour and nervous system in invertebrates. Neurosci Biobehav Rev.

[CR29] Freund N, Valencia-Alfonso CE, Kirsch J (2016). Asymmetric top-down modulation of ascending visual pathways in pigeons. Neuropsychologia.

[CR30] Golüke S, Bischof H-J, Engelmann J (2019). Social odour activates the hippocampal formation in zebra finches (*Taeniopygia guttata*). Behav Brain Res.

[CR31] Güntürkün O (1997). Avian visual lateralization: a review. NeuroReport.

[CR32] Güntürkün O, Ocklenburg S (2017). Ontogenesis of lateralization. Neuron.

[CR33] Güntürkün O, Hellmann B, Melsbach G, Prior H (1998). Asymmetries of representation in the visual system of pigeons. NeuroReport.

[CR34] Gusel’nikov VI, Morenkov ED, Hunh DC (1977). Responses and properties of receptive fields of neurons in the visual projection zone of the pigeon hyperstriatum. Neurosci Behav Physiol.

[CR35] Iwaniuk AN, Heesy CP, Hall MI, Wylie DRW (2008). Relative Wulst volume is correlated with orbit orientation and binocular visual field in birds. J Comp Physiol A Neuroethol Sens Neural Behav Physiol.

[CR36] Johnston M, Colombo M, Vonk J, Shackelford TK (2017). Entopallium. Encyclopedia of animal cognition and behavior.

[CR37] Johnston AN, Rogers LJ (1999). Light exposure of chick embryo influences lateralized recall of imprinting memory. Behav Neurosci.

[CR38] Johnston AN, Rogers LJ, Dodd PR (1995). [3H]MK-801 binding asymmetry in the IMHV region of dark-reared chicks is reversed by imprinting. Brain Res Bull.

[CR39] Karten HJ, Shimizu T (1989). The origins of neocortex: connections and lamination as distinct events in evolution. J Cogn Neurosci.

[CR40] Karten HJ, Hodos W, Nauta WJH, Revzin AM (1973). Neural connections of the “visual Wulst” of the avian telencephalon. Experimental studies in the pigeon (*Columba livia*) and owl (*Speotyto cunicularia*). J Comp Neurol.

[CR41] Keysers C, Diekamp B, Güntürkün O (2000). Evidence for physiological asymmetries in the intertectal connections of the pigeon (*Columba livia*) and their potential role in brain lateralisation. Brain Res.

[CR42] Knudsen EI (2020). Evolution of neural processing for visual perception in vertebrates. J Comp Neurol.

[CR43] Kuo ZY (1932). Ontogeny of embryonic behavior in aves. IV. The influence of embryonic movements upon the behavior after hatching. J Comp Psychol.

[CR44] Letzner S, Manns M, Güntürkün O (2020). Light-dependent development of the tectorotundal projection in pigeons. Eur J Neurosci.

[CR45] Lorenzi E, Mayer U, Rosa-Salva O, Vallortigara G (2017). Dynamic features of animate motion activate septal and preoptic areas in visually naïve chicks (*Gallus gallus*). Neuroscience.

[CR47] Lorenzi E, Mayer U, Rosa-Salva O (2019). Spontaneous and light-induced lateralization of immediate early genes expression in domestic chicks. Behav Brain Res.

[CR48] Manns M, Güntürkün O (1999). “Natural” and artificial monocular deprivation effects on thalamic soma sizes in pigeons. NeuroReport.

[CR49] Manns M, Güntürkün O (1999). Monocular deprivation alters the direction of functional and morphological asymmetries in the pigeon’s (*Columba livia*) visual system. Behav Neurosci.

[CR50] Manns M, Güntürkün O (2003). Light experience induces differential asymmetry pattern of GABA- and parvalbumin-positive cells in the pigeon’s visual midbrain. J Chem Neuroanat.

[CR51] Mascetti GG, Vallortigara G (2001). Why do birds sleep with one eye open? Light exposure of the chick embryo as a determinant of monocular sleep. Curr Biol.

[CR52] Mayer U, Pecchia T, Bingman VP (2016). Hippocampus and medial striatum dissociation during goal navigation by geometry or features in the domestic chick: an immediate early gene study. Hippocampus.

[CR53] Mayer U, Rosa-Salva O, Morbioli F, Vallortigara G (2017). The motion of a living conspecific activates septal and preoptic areas in naive domestic chicks (*Gallus gallus*). Eur J Neurosci.

[CR54] Mckenzie R, Andrew RJ, Jones RB (1998). Lateralization in chicks and hens: new evidence for control of response by the right eye system. Neuropsychologia.

[CR55] Medina L, Reiner A (2000). Do birds possess homologues of mammalian primary visual, somatosensory and motor cortices?. Trends Neurosci.

[CR56] Michael N, Löwel S, Bischof H-J (2015). Features of the retinotopic representation in the visual Wulst of a laterally eyed bird, the zebra finch (*Taeniopygia guttata*). PLoS ONE.

[CR57] Mihrshahi R (2006). The corpus callosum as an evolutionary innovation. J Exp Zool B.

[CR58] Morandi-Raikova A, Mayer U (2020). The effect of monocular occlusion on hippocampal c-Fos expression in domestic chicks (*Gallus gallus*). Sci Rep.

[CR59] Morandi-Raikova A, Danieli K, Lorenzi E (2021). Anatomical asymmetries in the tectofugal pathway of dark-incubated domestic chicks: rightwards lateralization of parvalbumin neurons in the entopallium. Laterality.

[CR60] Mouritsen H, Feenders G, Liedvogel M (2005). Night-vision brain area in migratory songbirds. Proc Natl Acad Sci USA.

[CR61] Ng BSW, Grabska-Barwińska A, Güntürkün O, Jancke D (2010). Dominant vertical orientation processing without clustered maps: early visual brain dynamics imaged with voltage-sensitive dye in the pigeon visual Wulst. J Neurosci.

[CR62] Nieder A, Wagner H (2000). Horizontal-disparity tuning of neurons in the visual forebrain of the behaving barn owl. J Neurophysiol.

[CR63] Parker DM, Delius JD (1972). Visual evoked potentials in the forebrain of the pigeon. Exp Brain Res.

[CR64] Pettigrew JD, Konishi M (1976). Neurons selective for orientation and binocular disparity in the visual Wulst of the barn owl (*Tyto alba*). Science.

[CR65] R Core Team (2020) R: a language and environment for statistical computing. R Foundation for Statistical Computing, Vienna, Austria. https://www.R-project.org/. Accessed 1 July 2020

[CR66] Rajendra S, Rogers LJ (1993). Asymmetry is present in the thalamofugal visual projections of female chicks. Exp Brain Res.

[CR67] Revzin AM (1969). A specific visual projection area in the hyperstriatum of the pigeon (*Columba livia*). Brain Res.

[CR68] Rogers LJ (1982). Light experience and asymmetry of brain function in chickens. Nature.

[CR69] Rogers LJ (1990). Light input and the reversal of functional lateralization in the chicken brain. Behav Brain Res.

[CR70] Rogers LJ (1997). Early experiential effects on laterality: research on chicks has relevance to other species. Laterality.

[CR71] Rogers LJ (2000). Evolution of hemispheric specialization: advantages and disadvantages. Brain Lang.

[CR72] Rogers L (2012). The two hemispheres of the avian brain: their differing roles in perceptual processing and the expression of behavior. J Ornithol.

[CR73] Rogers LJ, Bolden SW (1991). Light-dependent development and asymmetry of visual projections. Neurosci Lett.

[CR74] Rogers LJ, Deng C (1999). Light experience and lateralization of the two visual pathways in the chick. Behav Brain Res.

[CR75] Rogers LJ, Sink HS (1988). Transient asymmetry in the projections of the rostral thalamus to the visual hyperstriatum of the chicken, and reversal of its direction by light exposure. Exp Brain Res.

[CR76] Rogers LJ, Vallortigara G, Andrew RJ (2013). Divided brains: the biology and behaviour of brain asymmetries.

[CR77] Rosa Salva O, Regolin L, Vallortigara G (2007). Chicks discriminate human gaze with their right hemisphere. Behav Brain Res.

[CR78] Rosa Salva O, Regolin L, Vallortigara G (2012). Inversion of contrast polarity abolishes spontaneous preferences for face-like stimuli in newborn chicks. Behav Brain Res.

[CR79] Rugani R, Rosa Salva O, Regolin L, Vallortigara G (2015). Brain asymmetry modulates perception of biological motion in newborn chicks (*Gallus gallus*). Behav Brain Res.

[CR80] Shanahan M, Bingman VP, Shimizu T (2013). Large-scale network organization in the avian forebrain: a connectivity matrix and theoretical analysis. Front Comput Neurosci.

[CR81] Skiba M, Diekamp B, Güntürkün O (2002). Embryonic light stimulation induces different asymmetries in visuoperceptual and visuomotor pathways of pigeons. Behav Brain Res.

[CR82] Stacho M, Letzner S, Theiss C (2016). A GABAergic tecto-tegmento-tectal pathway in pigeons. J Comp Neurol.

[CR83] Stacho M, Herold C, Rook N (2020). A cortex-like canonical circuit in the avian forebrain. Science.

[CR84] Ströckens F, Freund N, Manns M (2013). Visual asymmetries and the ascending thalamofugal pathway in pigeons. Brain Struct Funct.

[CR85] Vallortigara G (1992). Right hemisphere advantage for social recognition in the chick. Neuropsychologia.

[CR86] Vallortigara G, Andrew RJ (1991). Lateralization of response by chicks to change in a model partner. Anim Behav.

[CR87] Vallortigara G, Andrew RJ (1994). Differential involvement of right and left hemisphere in individual recognition in the domestic chick. Behav Process.

[CR88] Vallortigara G, Rogers LJ (2005). survival with an asymmetrical brain: advantages and disadvantages of cerebral lateralization. Behav Brain Sci.

[CR89] Vallortigara G, Cozzutti C, Tommasi L, Rogers LJ (2001). How birds use their eyes: opposite left-right specialization for the lateral and frontal visual hemifield in the domestic chick. Curr Biol.

[CR90] Verhaal J, Kirsch JA, Vlachos I (2012). Lateralized reward-related visual discrimination in the avian entopallium. Eur J Neurosci.

[CR91] Watanabe S, Mayer U, Bischof H-J (2011). Visual Wulst analyses “where” and entopallium analyses “what” in the zebra finch visual system. Behav Brain Res.

[CR92] Wilson P (1980). The organization of the visual hyperstriatum in the domestic chick. II. Receptive field properties of single units. Brain Res.

[CR93] Zapka M, Heyers D, Hein CM (2009). Visual but not trigeminal mediation of magnetic compass information in a migratory bird. Nature.

